# Immune Activation Influences SAMHD1 Expression and Vpx-mediated SAMHD1 Degradation during Chronic HIV-1 Infection

**DOI:** 10.1038/srep38162

**Published:** 2016-12-06

**Authors:** Weihui Fu, Chao Qiu, Mingzhe Zhou, Lingyan Zhu, Yu Yang, Chenli Qiu, Linxia Zhang, Xuan Xu, Ying Wang, Jianqing Xu, Xiaoyan Zhang

**Affiliations:** 1 Shanghai Public Health Clinical Center, Institutes of Biomedical Sciences, Fudan University, Shanghai, China; 2 Key Laboratory of Medical Molecular Virology of Ministry of Education/Health at Shanghai Medical College, Fudan University, Shanghai, China; 3 Huashan Hospital, Fudan University, Shanghai, China; 4 Minhang Hospital, Fudan University, Shanghai, China; 5 Shanghai Municipal Center for Disease Control & Prevention, Shanghai, China

## Abstract

SAMHD1 restricts human immunodeficiency virus type 1 (HIV-1) replication in myeloid cells and CD4^+^ T cells, while Vpx can mediate SAMHD1 degradation to promote HIV-1 replication. Although the restriction mechanisms of SAMHD1 have been well-described, SAMHD1 expression and Vpx-mediated SAMHD1 degradation during chronic HIV-1 infection were poorly understood. Flow cytometric analysis was used to directly visualize *ex vivo*, and after *in vitro* SIV-Vpx treatment, SAMHD1 expression in CD4^+^ T cells and monocytes. Here we report activated CD4^+^ T cells without SAMHD1 expression were severely reduced, and SAMHD1 in CD4^+^ T cells became susceptible to SIV-Vpx mediated degradation during chronic HIV-1 infection, which was absent from uninfected donors. These alterations were irreversible, even after long-term fully suppressive antiretroviral treatment. Although SAMHD1 expression in CD4^+^ T cells and monocytes was not found to correlate with plasma viral load, Vpx-mediated SAMHD1 degradation was associated with indicators of immune activation. *In vitro* assays further revealed that T-cell activation and an upregulated IFN-I pathway contributed to these altered SAMHD1 properties. These findings provide insight into how immune activation during HIV-1 infection leads to irreparable aberrations in restriction factors and in subsequent viral evasion from host antiviral defenses.

SAMHD1 is an HIV-1 infection restriction factor. It potently restricts reverse transcription in myeloid cells and resting CD4^+^ T cells by hydrolyzing intracellular dNTPs or degrading newly synthesized viral RNA^1^,^2^,^3^,^4^, which is removable through DCAF1-CUL4/DDB1-E3 ubiquitin ligase complex mediated proteasome degradation upon simian immunodeficiency virus (SIV) derived Vpx treatment^5^. SAMHD1 is expressed abundantly in immune cells, including DC cells, B cells, monocytes and T cells^6^. Despite the reported inhibition of HIV-1 replication by numerous *in vitro* systems^6^,^7^,^8^,^9^, the relevance of SAMHD1 to HIV-1 pathogenesis *in vivo* remains controversial. Previous studies have demonstrated that HIV-1 elite controllers maintain higher levels of SAMHD1 transcripts than viraemic progressors do in PBMCs^10^,^11^. However, regulation of SAMHD1 is not found to correlate with viral load in SIV and HIV-2 infection models^12^,^13^. Therefore, the expression and distribution of SAMHD1 protein in subsets of HIV-1 target cells and its relationship with *in vivo* HIV replication in chronic HIV-1 infection need to be further investigated.

HIV-1 infection leads to chronic immune activation, functional impairment and gradual loss of CD4^+^ T cells, and ultimately Acquired Immune Deficiency Syndrome (AIDS) if combination antiretroviral therapy (cART) was not available^14^. Non-replicating HIV-1 virions can induce the activation of CD4^+^ T cells, and cause massive CD4^+^ T cells depletion by direct cell lysis and bystander apoptosis^15^,^16^. It is well known that activated CD4^+^ T cells are highly permissive to HIV-1 infection, whereas resting CD4^+^ T lymphocytes are refractory to HIV-1 infection. Interestingly, SAMHD1 restricts HIV-1 replication only in resting CD4^+^ T cells^6^, although SAMHD1 is abundantly expressed in activated CD4^+^ T cells as well^9^. In addition, upregulation of IFN-I pathway is one of markers that indicated persistent immune activation^17^. Sustained IFNα/β levels is associated with disease progression, and rapid progressors show stronger IFNα/β signatures than viraemic non-progressors^18^,^19^. SAMHD1 in monocytes is reported to be up-regulated by IFN-α^20^. However, another study has shown that SAMHD1 is induced poorly by IFN-α in monocytes and macrophages^21^. Since the regulation of SAMHD1 expression by immune activation is still obscure, we thus initiated experiments to investigate SAMHD1 expression in association with *in vivo* HIV-1 replication and immune activation during chronic HIV-1 infection.

## Results

### Characterization of SAMHD1 expression by flow cytometric analysis

**Figure 1:**
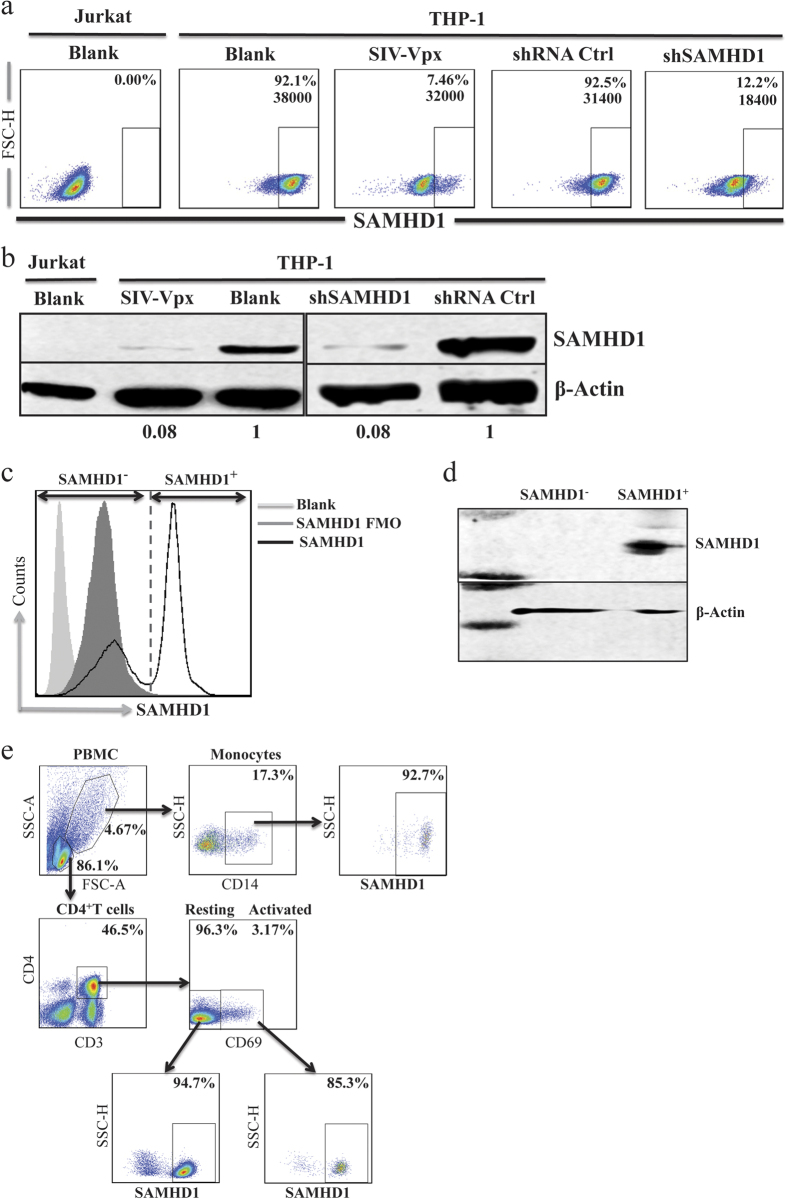
SAMHD1 expression can be reliably examined using flow cytometric analysis. (**a**) Dot-plots of SAMHD1 expression in THP-1 and Jurkat cell lines. THP-1 cells were treated with 4ng/mL SIV-Vpx for 24 h, or were untreated. SAMHD1-silent THP-1 cells (THP-1-shSAMHD1) or control shRNA (THP-1-shRNA Ctrl) were used as SAMHD1 staining controls. Percentages indicate percentage of SAMHD1^+^ cells, and digits represent MFI of SAMHD^+^ cells. **(b**) SAMHD1 expression was further confirmed using western blotting. Relative protein blot signal intensity analysis was performed using Li-Cor Image Studio software. Numbers represent relative value to the control or blank by normalization with to β-Actin. **(c**) PBMCs from an HIV-seronegative donor were stained with an antibody mixture with, or without, SAMHD1 pAb, a histogram illustrating SAMHD1 expression. (**d**) SAMHD1 positive and negative subgroups were sorted and expression of SAMHD1 was confirmed using western blotting. (**e**) Gating strategy results for SAMHD1 expression in activated CD4^+^ T cells, resting CD4^+^ T cells, and monocytes of PBMCs from an HIV-seronegative individual are presented. Percentages indicate percentage of SAMHD1^+^ cells.

We established a multicolor flow cytometric staining assay to contemporaneously determine SAMHD1 expression in different leukocyte subsets, including CD4^+^ T cells and monocytes. First, we used intracellular indirect immunofluorescence staining to evaluate SAMHD1 expression in cell lines. SAMHD1 expression was absent in Jurkat cells and present in most of the THP-1 cells (>92%), by 24 h of SIV-Vpx treatment, the percentage of THP-1 cells that expressed high level of SAMHD1 declined to 7.46% (Fig. 1a). We also used short hairpin RNAs (shRNA) to generate SAMHD1-silent (shSAMHD1-THP-1) and shRNA control THP-1 cells (shRNA Ctrl-THP-1). Compared with control cells, the percentage of SAMHD1 expressing cells decreased to 12.2% in the shSAMHD1-THP-1 cells. The mean fluorescence intensity (MFI) of SAMHD1 was also reduced (Fig. 1a). The results of flow cytometric analysis were validated in parallel using western blotting (Fig. 1b).

Next, PBMCs from healthy controls (HCs) were stained using a cocktail of antibodies for CD4^+^ T cells and monocytes, and were then analyzed using flow cytometry. The fluorescence signal results for SAMHD1 indicated that PBMCs distinctly divided into two subgroups. One subgroup overlapped with the Fluorescence Minus One (FMO) control with all antibodies from the cocktail except that against SAMHD1, and consisted of SAMHD1 negative (SAMHD1^-^) cells. The other subgroup with high fluorescence signal was defined as SAMHD1 positive cells (SAMHD1^+^) (Fig. 1c). Both subsets were sorted, and SAMHD1 expression was confirmed using western blotting (Fig. 1d). These results indicated that flow cytometric analysis can distinguish cells that express SAMHD1 from their negative counterparts. Because myeloid cells and CD4^+^ T cells from human peripheral blood are the primary target cells during HIV-1 infection, to this end, we examined SAMHD1 expression in these cells. Most of the CD14^+^ monocytes (CD14^+^Mo), activated CD4^+^ T cells (CD69^+^CD4^+^T), and resting CD4^+^ T cells (CD69^-^CD4^+^T) exhibited high SAMHD1 levels (Fig. 1e).

### SAMHD1 expression and Vpx-mediated SAMHD1 degradation in chronic HIV-1 infected individuals

**Figure 2:**
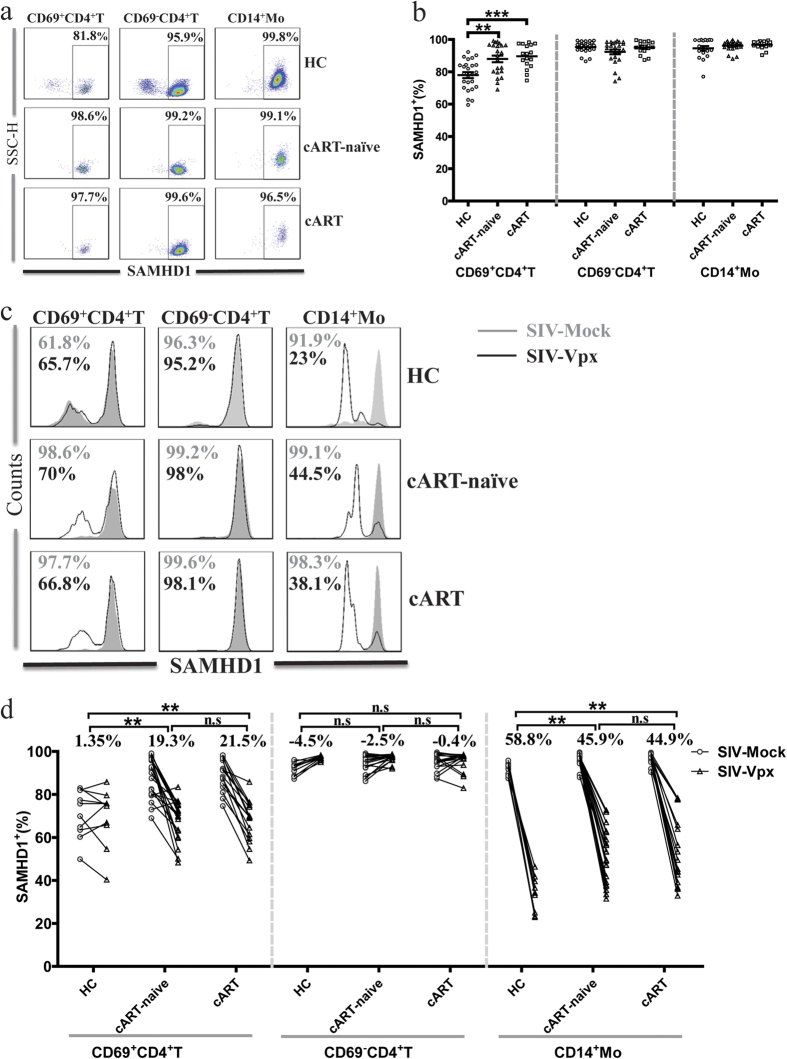
SAMHD1 expression and Vpx-mediated SAMHD1 degradation in HIV-1 target cells. **(a**) Representative flow cytometric analysis results for SAMHD1 expression in activated CD4^+^ T cells, resting CD4^+^ T cells, and monocytes from HC, cART-naive, and cART patients are presented. Numbers in top right indicate percent SAMHD1 positive cells. **(b**) Statistical analysis of data was obtained for *n* = 25 HC, *n* = 22 cART-naive individuals, or for *n* = 17 cART individuals. **(c**) Fresh isolated PBMCs were treated with 2 ng/mL SIV-Vpx or SIV-Mock for 48 h, and the expression of SAMHD1 in activated CD4^+^ T cells, resting CD4^+^ T cells, and monocytes from HC, cART-naive, and cART cohorts was determined. Representative histograms were shown. Numbers in top left indicate percent SAMHD1 positive cells in SIV-Mock (grey), and SIV-Vpx treatment (black), groups. **(d)** Shown are summary results for the effects of SIV-Vpx (“∆”) or SIV-Mock (“○”) on SAMHD1 expression in activated CD4^+^ T cells, resting CD4^+^ T cells, and monocytes from 10 HC, 22 cART-naive individuals, and 17 cART individuals. Numbers represent the mean value of loss of SAMHD1 positive cells after SIV-Vpx treatment. The significance was measured using the Mann-Whitney test, *p < 0.05, **p < 0.001, ***p < 0.0001; *n.s,* not significant.

HIV-1 mainly infected myeloid cells and CD4^+^ T cells, whether SAMHD1 expression in these subsets was influenced by HIV-1 remains unknown. To determine what happens to SAMHD1 expression during chronic HIV-1 infection, we employed this established flow cytometric method to analyze SAMHD1 expression in CD4^+^ T cells and CD14^+^ monocytes from HIV-1 positive patients who were naïve to cART (cART- naïve) and HIV-1-infected patients who received prolonged cART with undetectable plasma HIV-1 levels (referred as cART). Representative flow cytometry results are presented in Fig. 2a. No significant differences were found in the fraction of cells expressing SAMHD1 for resting CD4^+^ T cells and monocytes from cART naïve and cART-treated patients (Fig. 2b). However, compared with those from healthy controls, we found that most of the SAMHD1^-^ cells were absent from samples from infected patients, and this absence was not restored by cART and resulted in a higher percentage of SAMHD1^+^ in the remaining cells (Fig. 2c). The MFI of SAMHD1 in the remaining activated CD4^+^ T cells from the HIV-1 infected individuals was significantly higher compared with healthy donors (Supplementary Fig. S1).

*In vitro*, SIV-Vpx mediated SAMHD1 degradation in monocytes, macrophages, and dendritic cells. We examined whether HIV-1 infection resulted in changes in this SAMHD1 property in HIV-1 target cells. PBMCs were treated with SIV-Vpx for 48 h. The changes in SAMHD1 expression in these three cell subsets were examined using flow cytometric analysis. SIV-Vpx treatment decreased the SAMHD1^+^ cell frequency in monocytes and activated CD4^+^ T cells, but the SAMHD1^+^ cell frequency in resting CD4^+^ T cells was not affected (Fig. 2c). Loss of SAMHD1^+^ cells was most predominant in monocytes (58.8 ± 8.8%, 45.9 ± 12.5%, and 44.9 ± 14.9% in the HC, cART- naïve, and cART cohorts, respectively). This result indicated that in a proportion of monocytes, SAMHD1 became refractory to SIV-Vpx mediated degradation in chronic HIV-1 infection (Fig. 2d). In particular, a subset of activated CD4^+^ T cells from cART-naïve (19.3 ± 13.9%) and from cART (21.5 ± 7.9%) patients was susceptible to SIV-Vpx mediated SAMHD1 degradation. In contrast, SIV-Vpx treatment had no effect on SAMHD1 in activated CD4^+^ T cells from healthy donors (Fig. 2d). Decrease in SAMHD1 expression after SIV-Vpx treatment was not observed in resting CD4^+^ T cells (Fig. 2d). Consistent with the change in SAMHD1^+^ cell frequency, the levels of SAMHD1 also decreased in activated CD4^+^ T cells and monocytes, but not in resting CD4^+^ T cells (Supplementary Fig. S2). These data indicated HIV-1 infection resulted in increased SAMHD1 expression in activated CD4^+^ T cells. However, the sensitivity of SAMHD1 to SIV-Vpx in different cells was not consistent, monocytes were the most susceptible to Vpx- mediated degradation, activated CD4^+^ T cells were less, resting CD4^+^ T cells were the least.

### Association between SAMHD1 protein levels and *in vivo* HIV-1 replication

**Figure 3:**
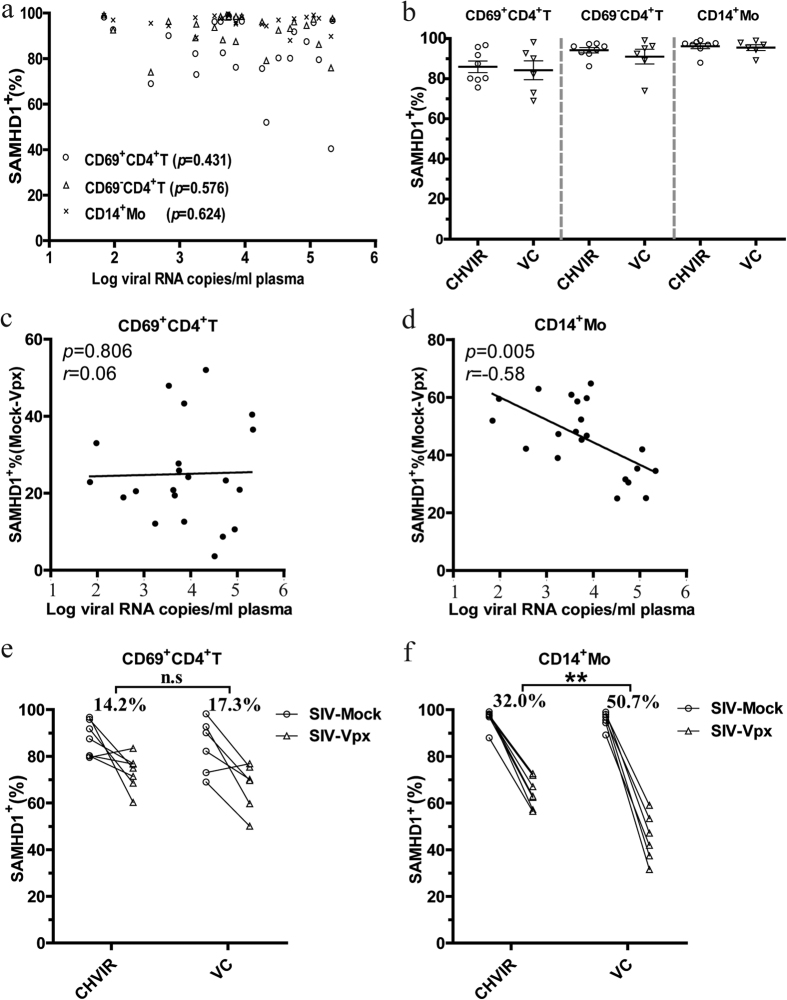
Relationship of SAMHD1 expression and degradation with *in vivo* HIV-1 replication. (**a**) Results of statistical analysis of the correlation between viral load and SAMHD1 expression in activated CD4^+^ T cells (“○”), resting CD4^+^ T cells (“▵”), or monocytes (“×”), in cART-naive patients (*n* = 22). Correlation statistics were analyzed using the Spearman correlation. *r*, Spearman’s correlation coefficient. **(b**) The proportions of SAMHD1 positive cells in activated CD4^+^ T cells, resting CD4^+^ T cells, and monocytes are presented for the chronic viremia (CHVIR, VL>10000 copies/mL), and viremic controllers (VC, VL<2000 copies/mL), cohorts, CHVIR, *n *= 8; VC, *n *= 6. Comparisons were made using the Mann-Whitney test, *p*-value is not indicated when p > 0.05. (**c**,**d**) There was no correlation for activated CD4^+^ T cells, whereas there was a negative correlation, between the frequency of cells susceptible to SAMHD1 degradation in monocytes and plasma viral loads from cART-naive subjects (*n* = 22). Correlation statistics were analyzed using the Spearman correlation. *r*, Spearman’s correlation coefficient. **(e**,**f**) The frequency of SAMHD1 degradation by SIV-Vpx in activated CD4^+^ T cells, and monocytes was compared between CHVIR and VC cohorts. Numbers represent the mean value of loss of SAMHD1 positive cells after SIV-Vpx treatment. *p* values were evaluated using the Mann-Whitney test, *p < 0.05, **p < 0.001, ***p < 0.0001; *n.s*, not significant. SIV-Vpx (“▵”), SIV-Mock (“○”).

Within the group of untreated HIV-1 infected patients, we next investigated whether SAMHD1 expression in these HIV-1 target cells correlated with plasma HIV-1 viral load, the laboratory indicator for *in vivo* HIV-1 replication. Because constitutively high SAMHD1 expression occurred in activated CD4^+^ T cells, resting CD4^+^ T cells, and monocytes, no significant correlations were observed between SAMHD1 expression and plasma viral load (Fig. 3a). To further illustrate this issue, we divided cART-naïve patients into chronic high viremia (CHVIR) with a viral load persistently >10000 copies per mL plasma and viremic controllers (VC) with a viral load persistently <2000 copies per mL plasma, and compared SAMHD1 levels of both groups. There were no significant differences in CD69^+^CD4^+^ T cells, CD69^-^CD4^+^ T cells, and monocytes (Fig. 3b). The between-group MFI analysis of SAMHD1^+^ cells revealed that there were statistically significant differences in resting CD4^+^ T cells, but the magnitude of the differences was slight (Supplementary Fig. S3). In conclusion, SAMHD1 expression in HIV-1 target cells was not associated with plasma viral load.

To assess whether aberrant SIV-Vpx mediated SAMHD1 degradation was associated with *in vivo* HIV-1 replication, we analyzed the relationship between the SAMHD1^+^ cell frequency susceptible to SIV-Vpx mediated degradation and plasma HIV-1 viral load. This correlation was not significant between the frequency of SAMHD1^+^ cells susceptible to SIV-Vpx mediated degradation in activated CD4^+^ T cells and viral load (Fig. 3c, *p* = 0.806). However, there was a significant negative correlation when CD14^+^ monocytes were examined (Fig. 3d, *p* = 0.005). In addition, SAMHD1^+^ cell frequency susceptible to SIV-Vpx mediated degradation in monocytes, but not in activated CD4^+^ T cells, was greater in the VC group compared with the CHVIR group (Fig. 3e and f). These data indicated the degradation of SAMHD1 mediated by SIV-Vpx in monocytes was related to plasma viral load, but it was not in activated CD4^+^ T cells.

### Association between SIV-Vpx mediated SAMHD1 degradation and predictors of HIV disease progression

**Figure 4:**
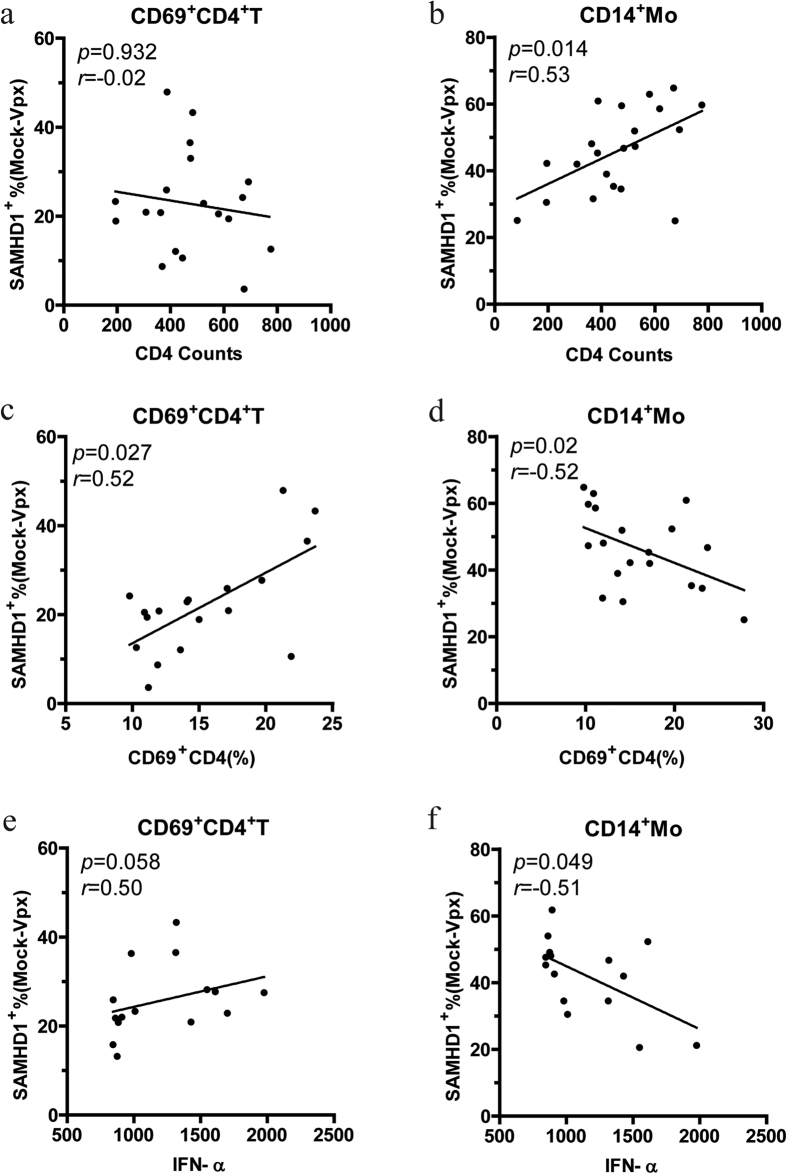
The correlation between Vpx-mediated SAMHD1 degradation and predictors of HIV disease in chronically HIV-1 infected patients. The relationship of SAMHD1 degradation by SIV-Vpx in activated CD4^+^ T cells with CD4 counts (**a**), the number of activated CD4^+^ T cells (**c**), or blood IFN-α level (**e**). Correlation between SAMHD1 degradation by SIV-Vpx in CD14^+^ monocytes and CD4^+^ T-cell counts (**b**), the number of activated CD4^+^ T cells (**d**), or blood IFN-α level (**f**). Subjects with CD4 counts and the number of activated CD4^+^ T cells were from cART-naive populations (*n* = 22); subjects with blood IFN-α level were from HIV-1 infected individuals (*n* = 15). *r*, Spearman’s correlation coefficient; *p *< 0.05 was considered to indicate a statistically significant result.

What factors can have an effect on Vpx-mediated SAMHD1 degradation except for plasma viral load? Next, we further investigated the relationship between SIV-Vpx mediated SAMHD1 degradation and HIV pathology, including CD4^+^ T-cell counts, T-cell activation, and IFN-I pathway up-regulation. We found that the SAMHD1^+^ cell frequency susceptible to SIV-Vpx mediated degradation in activated CD4^+^ T cells was positively correlated with the percentage of activated CD4^+^ T cells (Fig. 4c, *p* = 0.027), but neither with CD4^+^ T-cell counts (Fig. 4a, *p* = 0.932) nor with blood IFN-α levels (Fig. 4e, *p* = 0.058). In CD14^+^ monocytes, frequency of SAMHD1^+^ cells susceptible to SIV-Vpx mediated degradation was positively correlated with CD4^+^ T-cell count (Fig. 4b, *p* = 0.014), but was negatively correlated with the percentage of activated CD4^+^ T cells (Fig. 4d, *p* = 0.02) and with blood IFN-α levels (Fig. 4f, *p* = 0.049). These results showed that the Vpx-mediated SAMHD1 degradation in activated CD4^+^ T cells was associated with CD4^+^ T cells activation, while that in monocytes was influenced by multiple factors, including CD4 T cell count, IFN-α levels.

### Regulation of SAMHD1 by immune activation

**Figure 5:**
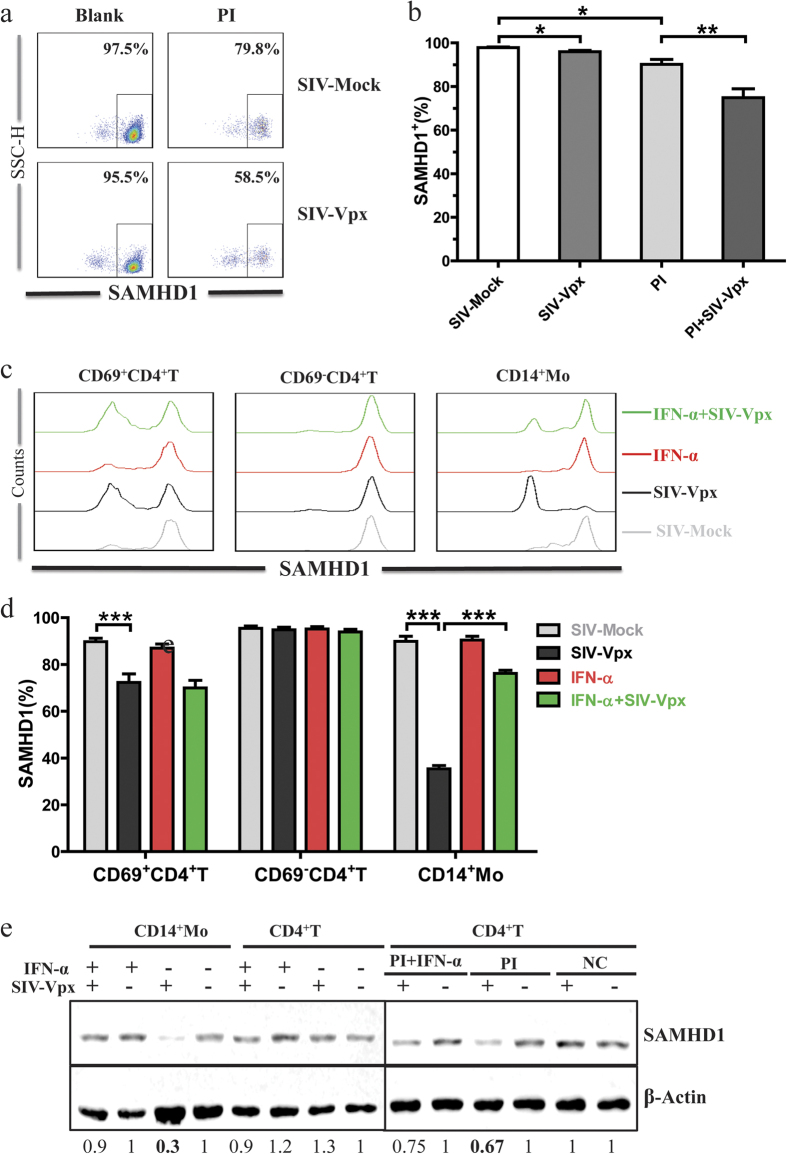
The effects of immune activation on SAMHD1 expression and SIV-Vpx mediated SAMHD1 degradation. (**a**) PBMC cells were stimulated with PI (1 μM PMA plus 1 μg/mL Ionomycin) for 24 h. Then, SAMHD1 expression in CD4^+^ T cells was assessed. Representative dot-plot results for SAMHD1 expression are presented. Percentages indicate absolute SAMHD1^+^ cell numbers. **(b**) Statistical analysis of data obtained as described in **a** was performed using the Mann-Whitney test. **(c)** PBMCs were pre-stimulated with IFN-α, or were not stimulated, for 24 h. Cells were then treated for another 48 h with SIV-Vpx or SIV-Mock. Histograms of SAMHD1 expression in activated CD4^+^ T cells, resting CD4^+^ T cells, and monocytes are presented. **(d**) Statistical analysis of data was performed using the Wilcoxon matched pairs test, data from HIV-1 infected individuals (n = 15). (**e**) Purified CD4^+^ T cells and monocytes were separated from PBMCs, pre-stimulated with PI or IFN-α, then treated with SIV-Vpx or SIV-Mock; whole cell lysates were examined by western blot analysis. Relative protein blot signal intensity analysis was performed using Li-Cor Image Studio software, numbers represent relative value normalized to β-Actin.

Above all, we see that SAMHD1 expression and SIV-Vpx mediated SAMHD1 degradation have been changed in chronic HIV-1 infection. As we all know, chronic HIV-1 infection is characterized by immune activation, which could regulate SAMHD1 expression *in vivo*^22^. To investigate whether SAMHD1 expression and degradation are associated with host immune activation, we first analyzed the effects of immune activation on SAMHD1 expression and Vpx-mediated degradation, in CD4^+^ T cells. Representative flow cytometry results are shown in Fig. 5a. Twenty-four hours after PMA plus Ionomycin stimulation, the SAMHD1^+^ CD4^+^ T-cell number decreased from 97.8% ± 0.7% to 90.2% ± 5.5%; SIV-Vpx treatment resulted in a further reduction, from 90.2% ± 5.5% to 74.8% ± 10.1% (Fig. 5b).

Next, we explored the effects of immune activation on SAMHD1 expression and Vpx- mediated loss of SAMHD1 in monocytes. Monocytes express a variety of Toll-like receptors (TLRs) and are susceptible to microbial products translocated through compromised gut mucosa^23^,^24^. We first examined the effects of stimulation of various TLR’s agonists on SAMHD1 expression and Vpx-mediated loss of SAMHD1 in monocytes. We found that TLR2, TLR3, TLR4, TLR7, and TLR8 agonists did not affect expression or SIV-Vpx mediated degradation of SAMHD1 (Supplementary Fig. S4).

The IFN-I pathway is activated during chronic HIV-1 infection, and SAMHD1 was reported as IFN stimulated gene^17^,^20^,^25^. We treated CD4^+^ T cells and monocytes with IFN-α, but SAMHD1 expression did not be changed in these cells (Supplementary Fig. S5). IFN-α did induce a dose-dependent inhibition of SIV-Vpx mediated SAMHD1 degradation in monocytes (Supplementary Fig. S6).

To further validate the role of IFN-α on SAMHD1 expression and degradation, we treated PBMCs from 15 HIV-1 infected individuals with IFN-α, or SIV-Vpx, or with a combination of IFN-α and SIV-Vpx. A representative flow cytometry result is presented in Fig. 5c. SAMHD1^+^ cells in monocytes were decreased by 55 ± 12.2% after SIV-Vpx treatment, and this decrease was blocked in the presence of IFN-α. In activated CD4^+^ T cells, the frequency of SAMHD1^+^ cells was reduced by 17.4 ± 9.3% after SIV-Vpx treatment. Stimulation with IFN-α had no effect on Vpx-mediated SAMHD1 degradation. In resting CD4^+^ T cells, the frequency of SAMHD1^+^ cells remained unchanged after SIV-Vpx or IFN-a treatment (Fig. 5d). Western blotting was used to further confirm these results in purified CD4^+^ T cells and monocytes (Fig. 5e).

These results suggest that HIV-1 infection-induced CD4^+^ T-cell activation explains why activated CD4^+^ T cells in chronically HIV-1 infected patients are susceptible to degradation by SIV-Vpx. Moreover, upregulation of the IFN-I pathway antagonized Vpx-mediated SAMHD1 degradation in monocytes.

## Discussion

We found aberrant alterations in SAMHD1 in HIV-1 target cells that could be explained by the immune activation caused by HIV-1 infection. HIV-1 infection leads to depletion of SAMHD1^-^ cells in activated CD4^+^ T cells. Long-term cART will not restore this cell subset. This result is consistent with the result of another study, which revealed that SAMHD1^-^ activated CD4^+^ T cells are more permissive to HIV-1 infection and thus might lead to the loss of this cell subset in HIV-1 infected patients^26^. SAMHD1 is a host enzyme that participates in the metabolism of DNA via regulation of intracellular dNTP levels^2^. Lower levels of SAMHD1 in activated CD4^+^ T cells may condition the cells susceptible to activation induced apoptosis or pyroptosis. Relatively higher levels of SAMHD1 would preserve the remaining activated CD4^+^ T cells from the inflammatory microenvironment present in chronically infected patients.

In PBMCs, HIV-1 elite controllers maintain higher levels of SAMHD1 transcripts than do viremic progressors^10^,^11^. We examined SAMHD1 expression at protein levels comparable to those of previous studies^12^,^13^. No evidence was found for an association between SAMHD1 expression and HIV-1 replication *in vivo*, given SAMHD1 is constitutively expressed by HIV-1 target cells. However, when we treated cells with SIV-Vpx, a subset of activated CD4^+^ T cells, but not resting CD4^+^ T cells, became susceptible to Vpx-mediated SAMHD1 degradation. A proportion of monocytes were refractory to SAMHD1 degradation by SIV-Vpx. Upon SIV-Vpx treatment, SAMHD1 was degraded via the DCAF1-CUL4/DDB1-E3 ubiquitin ligase complex. The biochemical mechanism involved in the differential susceptibility of SAMHD1 in primary cells remains unknown. There are several avenues for further investigations, including whether the cells express less of the ubiquitin ligase complex? Is SAMHD1 modified so that it can no longer interact with the ubiquitin ligase complex or with Vpx? Is the SAMHD1 in these various cell populations active or inactive to restrict HIV replication or metabolism of DNA?

There is no Vpx expression in HIV-1 or none of viral proteins from HIV-1 could lead to SAMHD1 degradation, thus the altered susceptibility of SAMHD1 to Vpx-mediated degradation observed in our study is caused by host factors rather than viral factors. We treated cells with Vpx and observed SAMHD1 degradation to evaluate the efficiency of machinery conducting SAMHD1 degradation. Our *in vitro* assay results suggested that cellular activation of CD4^+^ T cells promotes SAMHD1 degradation. The number of activated CD4^+^ T cells in HIV-1 infected patients was significantly higher than that of healthy controls, and the frequency of SAMHD1^+^ activated CD4^+^ T cells susceptible to SIV-Vpx mediated degradation was in proportion to the percentage of activated CD4^+^ T cells, but did not correlate with HIV-1 viral load. Therefore, SAMHD1 in SAMHD1^+^ CD4^+^ T cells became susceptible to SIV-Vpx mediated degradation during chronic HIV-1 infection. This result implied that the aberration of SAMHD1 in CD4^+^ T cells was caused by T-cell activation and may have contributed to loss of viral control.

In the HIV-1 infected patients, a proportion of monocytes was refractory to SAMHD1 degradation by SIV-Vpx. Microbial products translocated from a compromised gastrointestinal tract stimulate monocytes^23^. However, activation by microbial product has no effect on SAMHD1 degradation by SIV-Vpx in monocytes. Interestingly, we found that IFN-α specifically antagonized SAMHD1 degradation by SIV-Vpx in monocytes, but not in CD4^+^ T cells. In HIV-1 infected patients, activation of the IFN-I pathway is a strong disease progression predictor^27^. Taken together, these results indicate that *in vivo* IFN-I sensitized monocytes become refractory to SIV-Vpx mediated degradation in HIV-1 infected patients, which negatively correlates with HIV-1 viral load. Further study will reveal whether monocytes with SAMHD1 refractory to SIV-Vpx mediated degradation support HIV-1 replication.

After long-term suppression of HIV-1 replication by cART, these aberrations in SAMHD1 can’t be repaired. Many successfully treated HIV-1-infected individuals experience a syndrome characterized by increased T-cell activation and evidence of heightened inflammation and coagulation responses^28^. This residual immune dysregulation syndrome might be sufficient for maintaining the aberrations in SAMHD1. An alternative explanation is that long-term chronic immune activation may lead to irreparable dysfunctions in long lived CD4^+^ T cells or progenitors of myeloid lineage cells. In addition, SAMHD1 function is related to cell cycle, SAMHD1 protein is phosphorylated at residue T592 by CDK kinase^29^. We respectively stimulated CD4^+^ T cells and monocytes using PMA plus Ionomycin and IFN-α, and found that the transduction efficiency of SIV-Vpx was not influenced by cell activation and proliferation (Supplementary Fig. S7). However, SAMHD1 phosphorylation was regulated in CD4^+^ T cells upon activation by PMA plus Ionomycin, but not in monocytes treated with IFN-α (Supplementary Fig. S8). PMA plus Ionomycin activated CD4^+^ T cells significantly induced SAMHD1 phosphorylation at residue T592, and these phosphorylated SAMHD1 seemed not to be degraded by SIV-Vpx. This explained why SAMHD1 in activated CD4^+^ T cells is unable to inhibit HIV-1 replication. In conclusion, these data indicate that only non-phosphorylated SAMHD1 of activated CD4^+^ T cells, but not resting CD4^+^ T cells, can be degraded by SIV-Vpx. However, the difference of SIV-Vpx mediated SAMHD1 degradation between activated and resting CD4^+^ T cells needs to be further investigated.

In conclusion, due to chronic immune activation, the cells from chronic HIV-1 infected patients may have different characteristics with respect to SAMHD1 in comparison to healthy controls. Immune activation not only altered the expression of SAMHD1 in CD4^+^ T cells, but also affected the susceptibility of SAMHD1 to SIV-Vpx in activated CD4^+^ T cells and monocytes. It may link to the different potential to support HIV-1 replication in activated CD4^+^ T cells and resting CD4^+^ T cells. Whether the immune activation-induced rapid disease progression was related to the alteration of SAMHD1 requires further investigation. Finally, these data give us a hint that HIV-1 has evolved a mechanism that utilized the host’s microenvironment to escape from SAMHD1 restriction without antagonism of viral accessory protein. This mechanism would differ from those used by other restriction factors such as APOBEC3G^30^ and BST-2^31^. Future studies should focus on the mechanisms of effects of immune activation on SAMHD1 restriction of HIV-1 replication. SAMHD1 could also serve as a potential target for treating the immune activation that occurs during chronic HIV-1 infection.

## Methods

### Subjects

Subjects were recruited as previously described^32^,^33^. Thirty-nine subjects were HIV-1 infected individuals. Twenty-two of these were treatment naive to combination antiretroviral therapy (cART); 17 received cART that controlled the plasma viral load (VL) to undetectable levels. Twenty-five subjects were HIV-1-seronegative volunteers who were included as healthy controls (HC). The characteristics of the HIV-1 positive subjects are presented in Table 1. To evaluate the relevance of SAMHD1 expression with viremia, individuals were stratified into two groups: untreated HIV-1-infected subjects with chronic viremia (CHVIR, VL>10000 copies/mL) and viremic controllers (VC, VL<2000 copies/mL), just as shown in Table 2. Blood IFN-α mRNA levels (linear scale) were obtained from microarray data (GSE56837)^32^.

The study protocol was approved by the Ethical Committee of the Shanghai Public Health Clinical Center. The methods were carried out in accordance with the Declaration of Helsinki. Written informed consent was obtained from all participants.

### Cell Lines and Primary Cells

HEK 293 T cells were cultured in DMEM (Gibco) containing 2 mM L-glutamine, 10% fetal bovine serum, 50 U/mL penicillin, and 50 μg/mL streptomycin sulfate. THP-1 cells and Jurkat cells were cultured in RPMI-1640 medium (Gibco) supplemented with 10% FBS, penicillin (100 U/mL), streptomycin sulfate (100 mg/mL), and L-glutamine (2 mM). ShRNA silenced cell lines were generated via transduction of lentiviral vectors encoding nonspecific shRNA (pLKO-nontarget) or shRNA specific to SAMHD1 (shSAMHD1#2 TRCN-0000343808, sequence: CCGGGCCATCATCTTGGAATCCAAACTCGAGTTTGGATTCCAAGATGATG GCTTTTTG, Sigma). These cell lines were then selected for resistance to puromycin. Peripheral blood mononuclear cells (PBMCs) were isolated from fresh blood. CD14^+^ monocytes and CD4^+^ T cells were separated from PBMCs using the EasySep™ Human Monocyte and CD4^+^ T cells Enrichment Kit (StemCell Technologies), respectively. Flow cytometry results indicated that cell purity was >95%.

### Plasmids and Reagents

SIV3^+^Vpx^+^, SIV3^+^Vpx^-^, and VSV-G plasmids were provided by Andrea Cimarelli (Université de Lyon, Lyon, France). The lentiviral packaging plasmid psPAX2 was obtained from Addgene. LPS was derived from *Escherichia coli* O55:B5 (Sigma-Aldrich). Pam3CSK4, Poly IC HMW, ssRNA40, and R837 were purchased from InvivoGen.

### Cell Stimulation

Cells (10^6^) were stimulated with 1000 U/mL rIFN-α2b (Invitrogen), 1 μM PMA plus 1 μg/mL Ionomycin (Abcam), 1 μg/mL Pam3CSK4, 20 μg/mL poly IC HMW, 1 μg/mL lipopolysaccharide (LPS), 1 μg/mL R837, or 1 μg/mL ssRNA40 for 24 h. 2 ng/mL SIV-Vpx or SIV-Mock was used to treat cells for 48 h.

### Immunoblot Analysis

Cells were lysed in Pierce IP Lysis Buffer (Thermo Fisher) containing a 1% protease inhibitor cocktail (Sigma). Whole-cell lysates were resolved on SDS-PAGE, transferred to a 0.22 μm polyvinylidene fluoride membrane, and probed using rabbit anti-SAMHD1 polyclonal Ab (clone 1F9, Abcam), rabbit anti-phospho-SAMHD1 (Thr592) Antibody (CST), mouse anti–β-actin mAb (Santa Cruz), and corresponding horseradish peroxidase (HRP)-conjugated secondary antibodies (Santa Cruz). Vpx protein was detected by Anti-p24 (HIV-1/Clade A, B, C, Immune-Tech).

### Virus-Like Particle Production

Virus-like particles (VLPs) containing or lacking Vpx were produced from HEK 293 T cells co-transfected with SIV3^+^Vpx^+^ or SIV3^+^Vpx^-^, and VSV-G plasmids. The concentration of SIV-Vpx was measured using P27 ELISA (Biomerieux). For SAMHD1 shRNA virion packaging, SAMHD1 shRNA plasmids, psPAX, and pVSV-G were co-transfected into HEK 293 T cells.

### Flow Cytometry

Intracellular SAMHD1 staining was carried out as previously described^9^. Briefly, PBMCs were stained for 30 min at room temperature using a mixture of anti-CD14-APC, anti-CD69-APC-Cy7, and anti-CD3-PB antibodies. After the cells were washed, they were fixed for 30 min at room temperature using BD Cytofix Fixation Buffer. Cells were permeabilized in BD Phosflow Perm Buffer III for 2 min at 4 °C in the dark and then were washed twice. Cells were then stained for 1 h with rabbit anti-human SAMHD1 polyclonal antibodies (1:100, Proteintech), followed by a mixture of anti-CD4-PE, and donkey anti-rabbit Alexa Fluor 488, antibodies (1:200, Invitrogen), for an additional 1 h. After the washes, cells were detected using a BD FACSAria™ II Flow Cytometer. The antibodies used for flow cytometric analysis were PE-CD4 (Biolegend, PRA-T4), APC-CD14 (Biolegend, HCD14), Pacific Blue-CD3 (Biolegend, UCHT1), APC-Cy7-CD69 (Biolegend, FN50), rabbit anti-human SAMHD1 pAb (Proteintech, 12586-1-AP), and Alexa Fluor^®^ 488 donkey anti-rabbit IgG (H+L) (Invitrogen, A-21206). Data were analyzed using FlowJo 7.6 software.

### Statistical Analysis

Statistical analyses were performed using GraphPad Prism 5 software. The results were expressed as Mean  ±  Standard Error of the Mean (SEM) values. The Mann-Whitney test was used to compare between-group differences. Comparisons of SIV-Vpx treatment in the same sample was achieved using a paired sample *t-*test (Wilcoxon test). Correlations between variables were calculated using a Spearman rank correlation test. Differences were considered statistically significant when the *p*-value was<0.05 (*p < 0.05; **p < 0.01; ***p < 0.001).

## Additional Information

**How to cite this article**: Fu, W. *et al*. Immune Activation Influences SAMHD1 Expression and Vpx-mediated SAMHD1 Degradation during Chronic HIV-1 Infection. *Sci. Rep.* **6**, 38162; doi: 10.1038/srep38162 (2016).

**Publisher's note:** Springer Nature remains neutral with regard to jurisdictional claims in published maps and institutional affiliations.

## Supplementary Material

Supplementary Information

## Figures and Tables

**Table 1:** Clinical characteristics of subjects in HIV-1 infected cohorts.

	cART-naïve	cART
**No. of Cases**	**22**	**17**
**Infection Time**(Yars)	**8.4(6-10)**	**8.94(4-13)**
**Gender, n** (**%**)		
Males	**6(25%)**	**11(65%)**
Females	**18(75%)**	**6(35%)**
**Median Age** (years)	**48.5(35-57)**	**49.2(39-66)**
**CD4 T cell counting** (Mean, SEM)	**441.8 ± 35.56**	**571.7 ± 44.3**
**CD4/CD8 ratio** (Mean, SEM)	**0.38 ± 0.05**	**0.7 ± 0.09**
**Lg VL(copies/ml) (Mean, SEM)**	**3.92 ± 0.2**	**n.a.**
**Time on cART** (Years)	‒	**2.14(0.6-3.4)**

cART-naive, HIV-1 infected individuals naive to therapy; cART, individuals treated with cART and with a plasma HIV-1 RNA<50 copies/mL value at the time of cross-sectional evaluation; Infection duration, the time from diagnosis of HIV-1 infection to the time when sample was collected; Time on cART, the duration for which cART was received (all >6 months); SEM, standard error of the mean; n.a., not available; Age and Time presented as mean (range) values. CD4 absolute counts (cells/μL) were detected using the BD Trucount™ kit, plasma HIV-1 viral load (copies/mL) was examined using qualitative RNA assay. Results are presented as Mean** **±** **SEM values.

**Table 2:** Clinical characteristics of subjects in HIV-1 infected cART-naive cohorts.

	CHVIR	VC
**No. of Cases**	**8**	**6**
**Infection Time**(Yars)	**8.38(6**–**10)**	**8 (6–9)**
**Gender, n (%)**		
Males	**6(75%)**	**3(50%)**
Females	**2(25%)**	**3(50%)**
**Median Age (**years)	**51.4(35–57)**	**46(35–54)**
**CD4 T cell counting** (Mean, SEM)	**359.3 ± 63.8**	**453.2 ± 56.2**
**CD4/CD8 ratio** (Mean, SEM)	**0.25 ± 0.04**	**0.55 ± 0.09**
**Lg VL**(copies/ml) (Mean, SEM)	**4.8 ± 0.1**	**2.6 ± 0.2**

HIV-1 infected patients were divided into untreated HIV-1-infected subjects with chronic viremia (CHVIR, VL>10000copies/mL, n = 8) and viremic controllers (VC, VL<2000 copies/mL, n = 6). The characteristics of each group were presented.
