# Supplementary material for: Immune Activation Influences SAMHD1 Expression and Vpx-mediated SAMHD1 Degradation during Chronic HIV-1 Infection
Source: Sci Rep. 2016 Dec 6;6:38162. doi: 10.1038/srep38162 (PMC5138643; doi:10.1038/srep38162)
Supplement: Supplementary Information [file srep38162-s1.pdf]

Immune Activation Influences SAMHD1 Expression and Vpx-mediated SAMHD1 Degradation during Chronic HIV-1 Infection

Weihui Fu[1,2], Chao Qiu[1,2,3,4*], Mingzhe Zhou[1,2], Lingyan Zhu[2],Yu Yang[2], Chenli Qiu[2], Linxia Zhang[2], Xuan Xu[2], Ying Wang[5*], Jianqing Xu[1,2*], Xiaoyan Zhang[1,2*]

1. Shanghai Public Health Clinical Center, Institutes of Biomedical Sciences, Fudan University, Shanghai, China

2. Key Laboratory of Medical Molecular Virology of Ministry of Education/Health at Shanghai Medical College, Fudan University, Shanghai, China

3. Huashan Hospital, Fudan University, Shanghai, China

4. Minhang Hospital, Fudan University, Shanghai, China

5. Shanghai Municipal Center for Disease Control & Prevention, Shanghai, China

**These authors contributed equally to this study.*

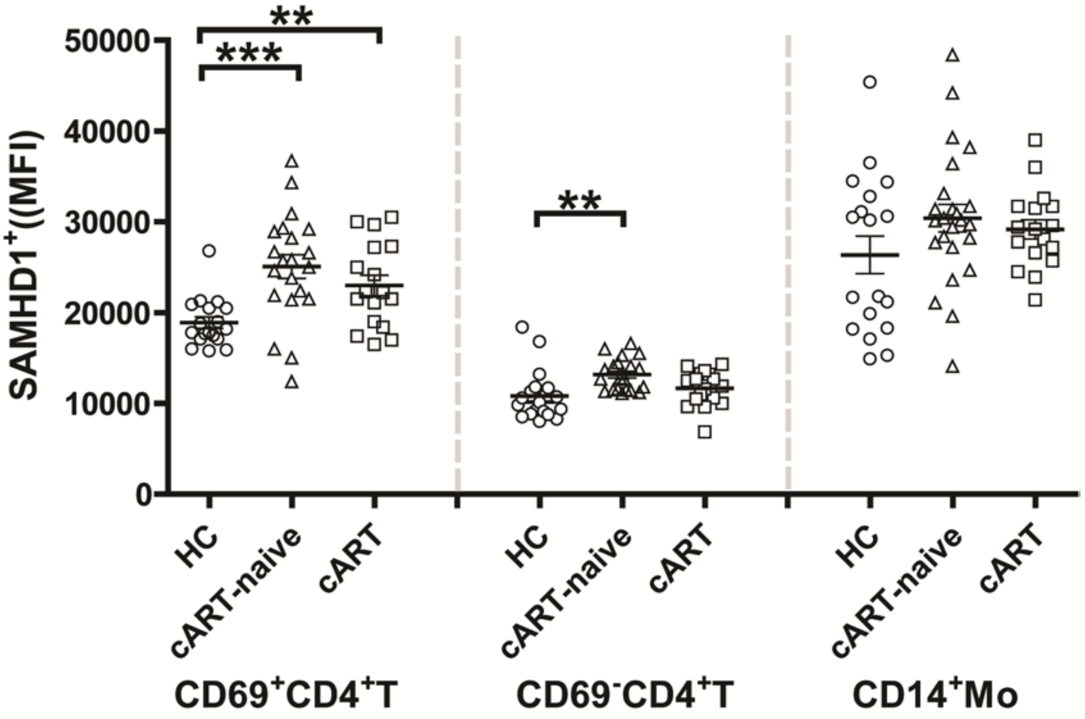

Supplementary Figure S1. The MFI of SAMHD1 expression in activated CD4$^+$ T cells, resting CD4$^+$ T cells, and monocytes of HIV-seronegative and HIV-1 infected individuals. The levels of SAMHD1 expression in activated CD4$^+$ T cells, resting CD4$^+$ T cells, and monocytes from HC (*n*=25), NAIVE subjects (*n*=22), or cART cohorts (*n*=17) are presented. The level of significance was measured using the Mann-Whitney test, *p<0.05, **p<0.001, ***p<0.0001. *p*-value is not indicated when p>0.05.

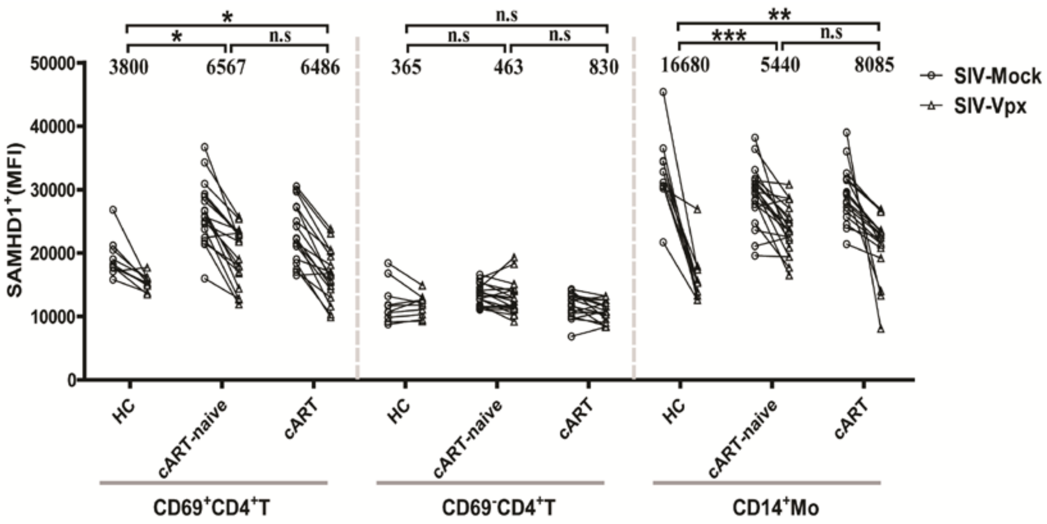

Supplementary Figure S2. The MFI of SAMHD1 degradation by SIV-Vpx in activated CD4^{+} T cells, resting CD4^{+} T cells, and monocytes of HIV-seronegative and HIV-1 infected individuals. The effects of SIV-Vpx on SAMHD1 levels in monocytes, activated CD4^{+} T cells, and resting CD4^{+} T cells, from 10 HC, 22 NAIVE individuals, and 17 cART individuals. The numbers represent the mean values for SAMHD1 loss (SAMHD1 MFI in Ctrl group minus that in SIV-Vpx treatment group, for each donor); the level of significance was measured using the Mann-Whitney test, *p<0.05, **p<0.001, ***p<0.0001; *n.s*, not significant. SIV-Vpx (“Δ”), SIV-Mock (“○”)

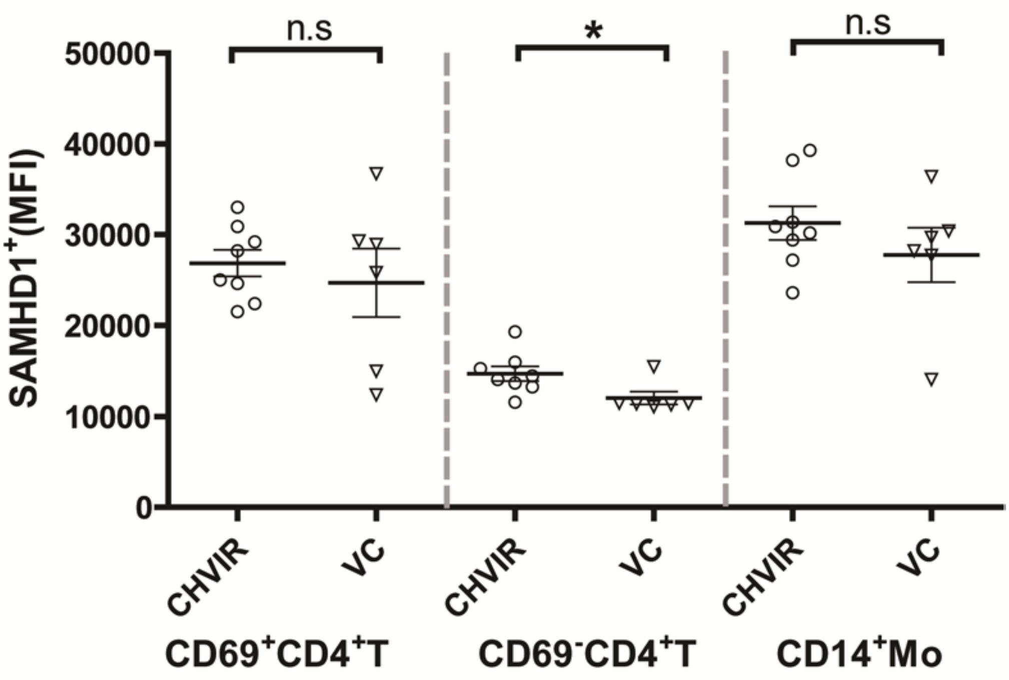

Supplementary Figure S3. The levels of SAMHD1 positive cells in activated CD4$^+$ T cells, resting CD4$^+$ T cells, and monocytes between CHVIR and VC cohorts. CHVIR, *n*=8; VC, *n*=6. Comparisons were made using the Mann-Whitney test, *p<0.05, **p<0.001, ***p<0.0001, *n.s*, not significant.

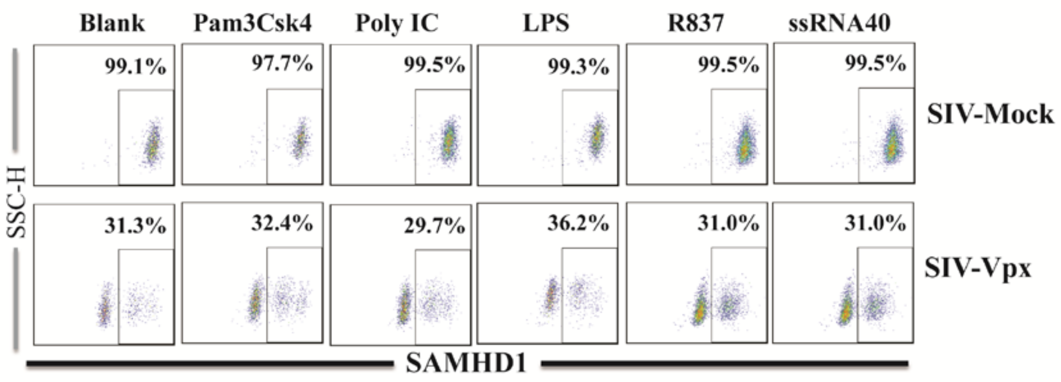

Supplementary Figure S4. TLR agonists had no effect on SAMHD1 expression and SIV-Vpx mediated SAMHD1 degradation in monocytes. PBMCs were pre-stimulated with culture media, PamCSK4 (1μg/mL), poly IC (20μg/mL), LPS (1μg/mL), R837 (1μg/mL), or ssRNA40 (1μg/mL) for 24 h; cells were then treated for another 48 h with SIV-Vpx or SIV-Mock; SAMHD1 expression in monocytes was detected using flow cytometry. Numbers in top right indicate percent SAMHD1 positive cells. Results for a representative experiment are presented.

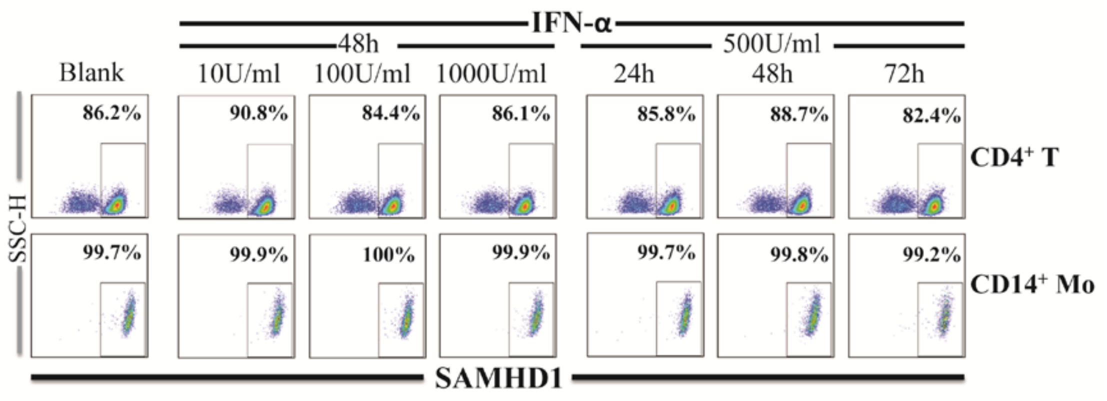

Supplementary Figure S5. SAMHD1 expression was not regulated by IFN-α. PBMCs from a healthy donor were stimulated with IFN-α at different concentrations and for different time periods; SAMHD1 expression was detected in $CD4^+$ T cells and monocytes.

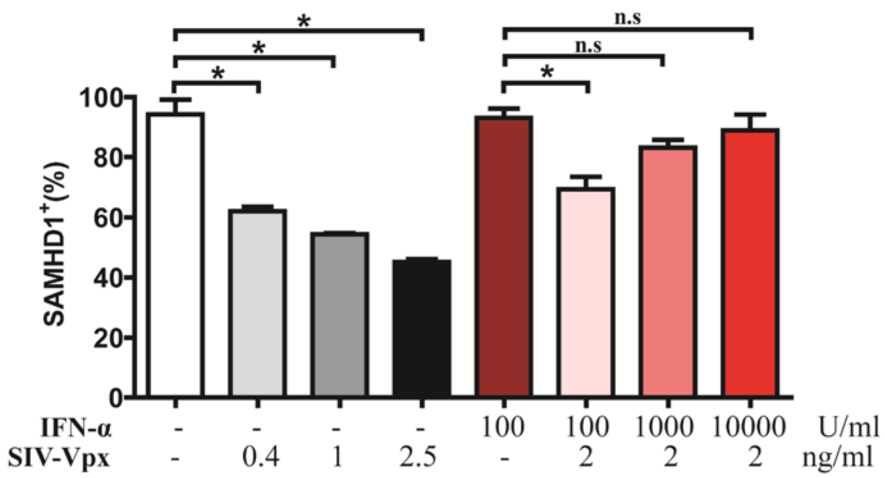

Supplementary Figure S6. Antagonism of Vpx-mediated SAMHD1 degradation in monocytes was IFN-α concentration dependent. PBMCs were pre-stimulated with IFN-α, or were unstimulated, for 24 h; cells were then treated for an additional 48 h with SIV-Vpx or SIV-Mock and the percentage of SAMHD1 was determined (*n*=3). *p* values were calculated using the Wilcoxon matched pairs test, *p<0.05, **p<0.001, ***p<0.0001.

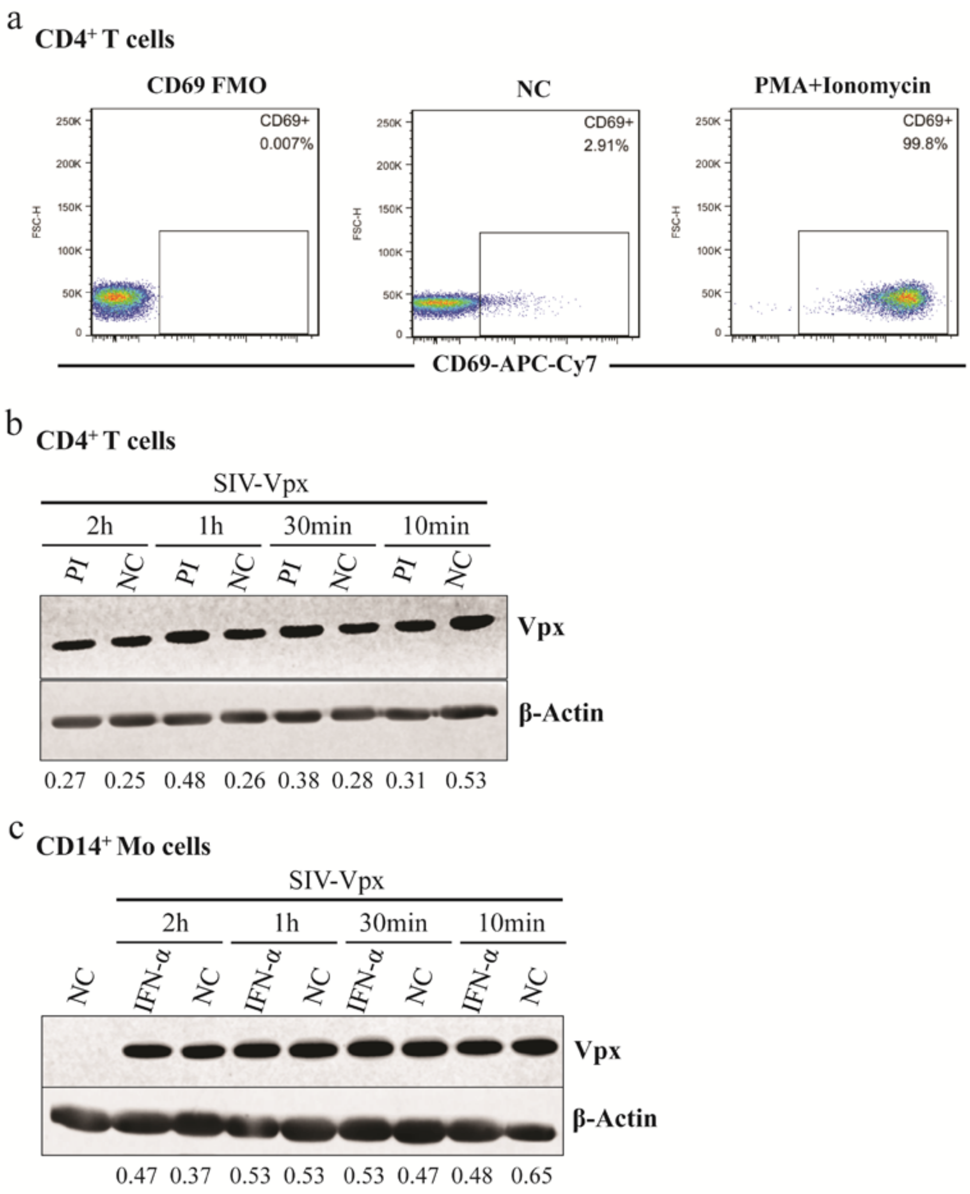

Supplementary Figure S7. There was no obvious difference in the transduction efficiency of SIV-Vpx between activated CD4$^+$ T /Mo cells and resting CD4$^+$ T/Mo cells. CD4$^+$ T cells from healthy donor were sorted and stimulated with PI (1μM PMA plus 1μg/mL Ionomycin) for 24h, and CD69 expression was measured by flow cytometry **(a)**. Then, SIV-Vpx was added for indicated time, whole cell lysates were examined by western blot analysis **(b)**. CD14$^+$ monocytes from healthy donor were sorted and stimulated with IFN-α for 24h, then, SIV-Vpx was added for indicated time, and whole cell lysates were examined by western blot analysis **(c).** Numbers represent value of Vpx related to β-Actin.

a

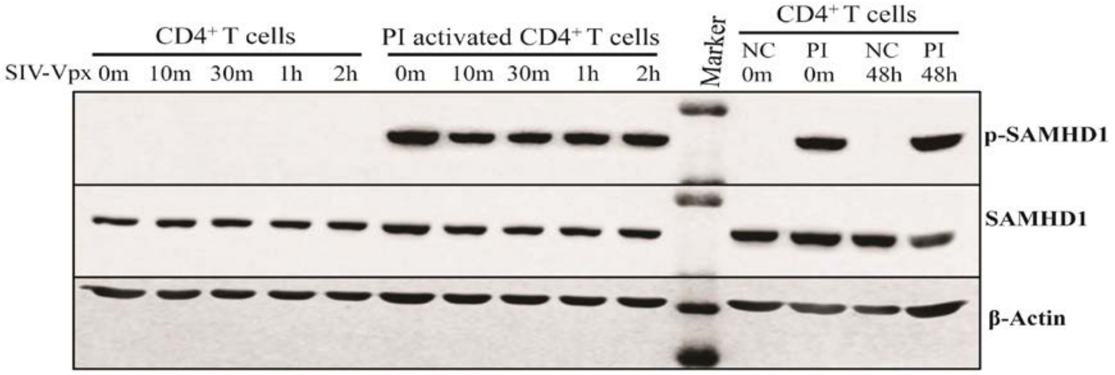

b

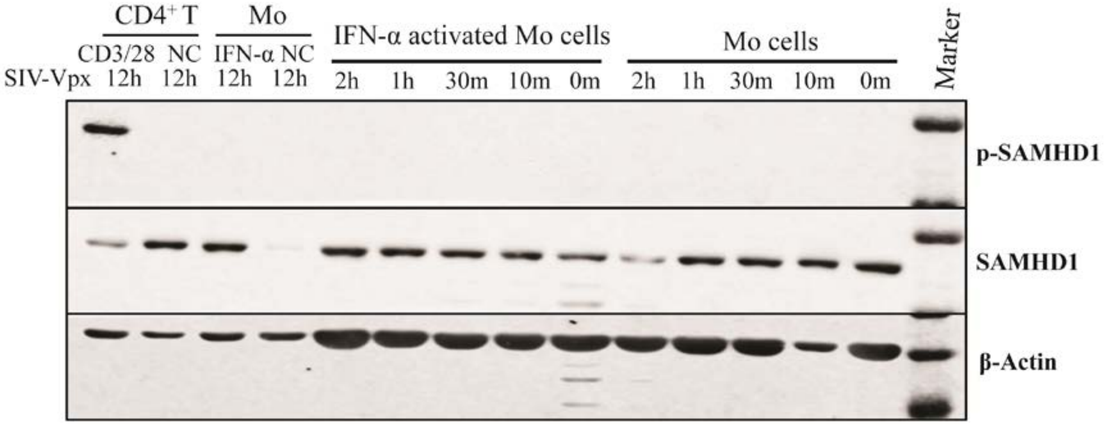

Supplementary Figure S8. Detection of SAMHD1 phosphorylation at T592 site between activated CD4$^+$ T /Mo cells and resting CD4$^+$ T/Mo cells in the presence of Vpx or not. **(a)** CD4$^+$ T cells from healthy donor were sorted and stimulated with PI (1μM PMA plus 1μg/mL Ionomycin) for 24h, Then, SIV-Vpx was added for indicated time, and whole cell lysates were examined by western blot analysis. **(b)** CD14$^+$ monocytes from healthy donor were sorted and stimulated with IFN-α for 24h, then, SIV-Vpx was added for indicated time, and whole cell lysates were examined by western blot analysis. CD3/28 stimulated CD4$^+$ T cells were used a positive control for SAMHD1 phosphorylation.
